# Multiple genome analysis of *Candida glabrata* clinical isolates renders new insights into genetic diversity and drug resistance determinants

**DOI:** 10.15698/mic2022.11.786

**Published:** 2022-10-13

**Authors:** Pedro Pais, Mónica Galocha, Azusa Takahashi-Nakaguchi, Hiroji Chibana, Miguel C. Teixeira

**Affiliations:** 1 Department of Bioengineering, Instituto Superior Técnico, Universidade de Lisboa, Lisbon, Portugal.; 2 iBB - Institute for Bioengineering and Biosciences, Biological Sciences Research Group, Instituto Superior Técnico, Lisboa, Portugal.; 3 Associate Laboratory i4HB—Institute for Health and Bioeconomy at Instituto Superior Técnico, Universidade de Lisboa, Portugal.; 4 Medical Mycology Research Center (MMRC), Chiba University, Chiba, Japan.

**Keywords:** Candida glabrata, clinical isolates, SNP, CNV, genome variation, drug resistance

## Abstract

The emergence of drug resistance significantly hampers the treatment of human infections, including those caused by fungal pathogens such as *Candida* species. *Candida glabrata* ranks as the second most common cause of candidiasis worldwide, supported by rapid acquisition of resistance to azole and echinocandin antifungals frequently prompted by single nucleotide polymorphisms (SNPs) in resistance associated genes, such as *PDR1* (azole resistance) or *FKS1/2* (echinocandin resistance). To determine the frequency of polymorphisms and genome rearrangements as the possible genetic basis of *C. glabrata* drug resistance, we assessed genomic variation across 94 globally distributed isolates with distinct resistance phenotypes, whose sequence is deposited in GenBank. The genomes of three additional clinical isolates were sequenced, in this study, including two azole resistant strains that did not display Gain-Of-Function (GOF) mutations in the transcription factor encoding gene *PDR1*. Genomic variations in susceptible isolates were used to screen out variants arising from genome diversity and to identify variants exclusive to resistant isolates. More than half of the azole or echinocandin resistant isolates do not possess exclusive polymorphisms in *PDR1* or *FKS1/2*, respectively, providing evidence of alternative genetic basis of antifungal resistance. We also identified copy number variations consistently affecting a subset of chromosomes. Overall, our analysis of the genomic and phenotypic variation across isolates allowed to pinpoint, in a genome-wide scale, genetic changes enriched specifically in antifungal resistant strains, which provides a first step to identify additional determinants of antifungal resistance. Specifically, regarding the newly sequenced strains, a set of mutations/genes are proposed to underlie the observed unconventional azole resistance phenotype.

## INTRODUCTION

Pathogenic yeasts from the *Candida* genus represent one of the most frequent causes of opportunistic fungal infections, associated with high morbidity and mortality worldwide [1]. Among *Candida* species, *Candida albicans* is the most common causative agent of infections in humans but can be successfully treated with current antifungal agents. On the other hand, the second most common etiological agent of systemic candidiasis – *Candida glabrata* – rapidly acquires resistance during antifungal therapy with azole antifungals and the emergence of clinical isolates resistant to the more recent echinocandin antifungals is on the rise [1, 2].

Clinical acquisition of azole or echinocandin resistance in *C. glabrata* is generally attributed to the acquisition of Single Nucleotide Polymorphisms (SNPs) in a very limited number of resistance associated genes. Gain-of-Function (GOF) mutations in the transcription factor (TF) *PDR1* result in protein hyperactivity and constitutive overexpression of multidrug resistance transporters, responsible for azole drug extrusion [3–7]. Resistance to echinocandins is based on point mutations in hotspot regions of the drug target protein encoded by *FKS1/2* genes, leading to decreased drug affinity to its target [8, 9]. Genome plasticity is a transversal trait to the clinically relevant *Candida* pathogens and the acquisition of polymorphisms is especially prominent in *C. glabrata* due to its haploid nature. Therefore, the determination of genetic variations observed in the genomes of resistant clinical isolates is a powerful tool to clarify the emergence of antifungal drug resistance.

This study makes use of genome sequencing data from 97 *C. glabrata* isolates collected from distinct geographical regions and body sites. All the analyzed isolates have known antifungal susceptibility profiles, which were used together with the genomic data to ascertain which genetic variations are more commonly associated with drug resistance phenotypes. Other than probing the frequency of variants in established resistance genes, this study also highlights a significant fraction of resistant isolates showing no genomic variations in well-known resistance determinants. The results imply that additional resistance mechanisms do arise in the clinical setting and pinpoint further genomic variants potentially leading to the emergence of resistance phenotypes. Differential genomic variations in isolates resistant to distinct drugs were also catalogued, allowing to extract core sets of genome changes associated with specific phenotypes. The possible role in antifungal drug resistance played by a set of genes whose mutations were associated with drug resistant clinical isolates was assessed. Optimally, the conclusions from this study contribute to guide the exploitation of additional genetic basis of antifungal drug resistance and identify new candidate genes as resistant determinants in *C. glabrata*.

## RESULTS AND DISCUSSION

### Genomic variation landscape across *C. glabrata* isolates exhibiting a large range of antifungal drug susceptibilities

**Figure 1:**
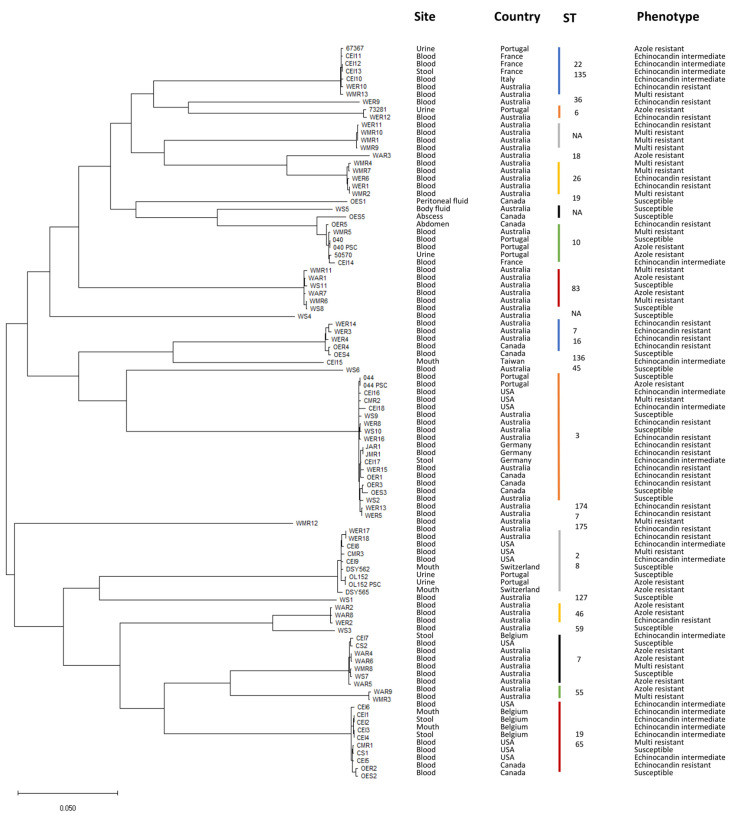
FIGURE 1: Isolates phylogeny. Phylogenetic tree representing evolutionary distances among the 97 *C. glabrata* isolates analyzed in this study. Phylogeny was inferred using CSI Phylogeny 1.4 [51] and tree representation was generated with MEGA [52]. ST – Sequence Type.

This study analyzes the genomes of 97 *C. glabrata* clinical isolates, collected from ten different countries, with known susceptibility phenotypes to azoles and echinocandins (**Table 1**). The phylogenetic relationship among these strains was inferred, yielding clades grouped according to distinct sequence types (**Figure 1**). Phenotypic profiles were used to classify each strain as drug susceptible (22.6%), echinocandin intermediate (18.5%), echinocandin resistant (23.7%), or azole (17.5%) resistant (**Table 2**). Isolates displaying multi resistance to antifungal drugs are also considered. From the 17 multi resistant isolates (17.5%), 16 are resistant to azoles and echinocandins, while one is resistant to amphotericin B and echinocandins (**Table 2**). This categorization was used to establish distinct resistance groups: susceptible, echinocandin intermediate, echinocandin resistant, azole resistant, and multi resistant.

**Table 1.** Data on the 97 *C. glabrata* isolates analyzed in this study.

**Isolate**	**Ref ID**	**ST**	**Collection site**	**Country**	**Phenotype**	**Reference**
**50570**	50570	10	Urine	Portugal	Azole resistant	This study
**67367**	67367	22	Urine	Portugal	Azole resistant	This study
**73281**	73281	6	Urine	Portugal	Azole resistant	This study
**040**	040	10	Blood	Portugal	Susceptible	[41]
**040_PSC**	040_PSC	10	Blood	Portugal	Azole resistant	Submitted for publication
**044**	044	3	Blood	Portugal	Susceptible	[41]
**044_PSC**	044_PSC	3	Blood	Portugal	Azole resistant	Submitted for publication
**CEI1**	B1012M	65	Mouth	Belgium	Echinocandin intermediate	[47]
**CEI10**	EI1815Blo1	22	Blood	Italy	Echinocandin intermediate	[47]
**CEI11**	EF1237Blo1	22	Blood	France	Echinocandin intermediate	[47]
**CEI12**	EF0616Blo1	135	Blood	France	Echinocandin intermediate	[47]
**CEI13**	EF1620Sto	135	Stool	France	Echinocandin intermediate	[47]
**CEI14**	EF1535Blo1	10	Blood	France	Echinocandin intermediate	[47]
**CEI15**	P35_2	136	Mouth	Taiwan	Echinocandin intermediate	[47]
**CEI16**	CST110	3	Blood	USA	Echinocandin intermediate	[47]
**CEI17**	EG01004Sto	3	Stool	Germany	Echinocandin intermediate	[47]
**CEI18**	BG2	3	Blood	USA	Echinocandin intermediate	[47]
**CEI2**	B1012S	65	Stool	Belgium	Echinocandin intermediate	[47]
**CEI3**	EB101M	19	Mouth	Belgium	Echinocandin intermediate	[47]
**CEI4**	BO101S	19	Stool	Belgium	Echinocandin intermediate	[47]
**CEI5**	CST34	19	Blood	USA	Echinocandin intermediate	[47]
**CEI6**	CST109	19	Blood	USA	Echinocandin intermediate	[47]
**CEI7**	EB0911Sto1	7	Stool	Belgium	Echinocandin intermediate	[47]
**CEI8**	M12	8	Blood	USA	Echinocandin intermediate	[47]
**CEI9**	CST78	8	Blood	USA	Echinocandin intermediate	[47]
**CMR1**	M7	19	Blood	USA	Multi resistant	[47]
**CMR2**	M6	3	Blood	USA	Multi resistant	[47]
**CMR3**	M7	8	Blood	USA	Multi resistant	[47]
**CS1**	CST80	19	Blood	USA	Susceptible	[47]
**CS2**	CST35	7	Blood	USA	Susceptible	[47]
**DSY562**	DSY562	8	Mouth	Switzerland	Susceptible	[48]
**DSY565**	DSY565	8	Mouth	Switzerland	Azole resistant	[48]
**JAR1**	NRZ_2016_57	3	Blood	Germany	Echinocandin resistant	[45]
**JMR1**	NRZ_2016_58	3	Blood	Germany	Echinocandin resistant	[45]
**OER1**	SP3003	3	Blood	Canada	Echinocandin resistant	[44]
**OER2**	SP3439	19	Blood	Canada	Echinocandin resistant	[44]
**OER3**	SP3689	3	Blood	Canada	Echinocandin resistant	[44]
**OER4**	SP2659	16	Blood	Canada	Echinocandin resistant	[44]
**OER5**	SP1643	10	Abdomen	Canada	Echinocandin resistant	[44]
**OES1**	SP2982	19	Peritoneal fluid	Canada	Susceptible	[44]
**OES2**	SP3046	19	Blood	Canada	Susceptible	[44]
**OES3**	SP3417	3	Blood	Canada	Susceptible	[44]
**OES4**	SP2320	16	Blood	Canada	Susceptible	[44]
**OES5**	SP1533	NA	Abscess	Canada	Susceptible	[44]
**OL152**	OL152	2	Urine	Portugal	Susceptible	[41]
**OL152_PSC**	OL152_PSC	2	Urine	Portugal	Azole resistant	[60]
**WAR1**	WM_18.48	83	Blood	Australia	Azole resistant	[46]
**WAR2**	WM_18.51	46	Blood	Australia	Azole resistant	[46]
**WAR3**	WM_05.113	18	Blood	Australia	Azole resistant	[46]
**WAR4**	WM_18.33	7	Blood	Australia	Azole resistant	[46]
**WAR5**	WM_04.387	7	Blood	Australia	Azole resistant	[46]
**WAR6**	WM_04.242	7	Blood	Australia	Azole resistant	[46]
**WAR7**	WM_03.419	83	Blood	Australia	Azole resistant	[46]
**WAR8**	WM_18.40	46	Blood	Australia	Azole resistant	[46]
**WAR9**	WM_18.62	55	Blood	Australia	Azole resistant	[46]
**WER1**	WM_18.54	26	Blood	Australia	Echinocandin resistant	[46]
**WER10**	WM_18.37	22	Blood	Australia	Echinocandin resistant	[46]
**WER11**	WM_18.41	NA	Blood	Australia	Echinocandin resistant	[46]
**WER12**	WM_18.38	6	Blood	Australia	Echinocandin resistant	[46]
**WER13**	WM_18.55	174	Blood	Australia	Echinocandin resistant	[46]
**WER14**	WM_18.57	16	Blood	Australia	Echinocandin resistant	[46]
**WER15**	WM_18.59	3	Blood	Australia	Echinocandin resistant	[46]
**WER16**	WM_18.60	3	Blood	Australia	Echinocandin resistant	[46]
**WER17**	WM_18.63	8	Blood	Australia	Echinocandin resistant	[46]
**WER18**	WM_18.64	8	Blood	Australia	Echinocandin resistant	[46]
**WER2**	WM_18.47	46	Blood	Australia	Echinocandin resistant	[46]
**WER3**	WM_18.52	16	Body fluid	Australia	Echinocandin resistant	[46]
**WER4**	WM_18.24	7	Blood	Australia	Echinocandin resistant	[46]
**WER5**	WM_18.30	7	Blood	Australia	Echinocandin resistant	[46]
**WER6**	WM_18.67	26	Tissue	Australia	Echinocandin resistant	[46]
**WER7**	WM_03.707	83	Blood	Australia	Echinocandin resistant	[46]
**WER8**	WM_04.194	3	Blood	Australia	Echinocandin resistant	[46]
**WER9**	WM_18.36	36	Blood	Australia	Echinocandin resistant	[46]
**WMR1**	WM_18.53	NA	Blood	Australia	Multi resistant	[46]
**WMR10**	WM_18.42	NA	Blood	Australia	Multi resistant	[46]
**WMR11**	WM_18.65	83	Blood	Australia	Multi resistant	[46]
**WMR12**	WM_18.66	175	Blood	Australia	Multi resistant	[46]
**WMR13**	WM_18.56	22	Blood	Australia	Multi resistant	[46]
**WMR2**	WM_18.49	26	Blood	Australia	Multi resistant	[46]
**WMR3**	WM_05.111	55	Blood	Australia	Multi resistant	[46]
**WMR4**	WM_18.27	26	Blood	Australia	Multi resistant	[46]
**WMR5**	WM_18.26	10	Blood	Australia	Multi resistant	[46]
**WMR6**	WM_03.450	83	Blood	Australia	Multi resistant	[46]
**WMR7**	WM_03.449	26	Blood	Australia	Multi resistant	[46]
**WMR8**	WM_03.698	7	Blood	Australia	Multi resistant	[46]
**WMR9**	WM_04.113	NA	Blood	Australia	Multi resistant	[46]
**WS1**	WM_18.45	127	Blood	Australia	Susceptible	[46]
**WS10**	WM_18.35	3	Blood	Australia	Susceptible	[46]
**WS11**	WM_18.39	83	Blood	Australia	Susceptible	[46]
**WS2**	WM_18.44	3	Blood	Australia	Susceptible	[46]
**WS3**	WM_18.50	59	Blood	Australia	Susceptible	[46]
**WS4**	WM_05.155	NA	Blood	Australia	Susceptible	[46]
**WS5**	WM_18.29	NA	Body fluid	Australia	Susceptible	[46]
**WS6**	WM_18.31	45	Blood	Australia	Susceptible	[46]
**WS7**	WM_03.308	7	Blood	Australia	Susceptible	[46]
**WS8**	WM_18.43	83	Blood	Australia	Susceptible	[46]
**WS9**	WM_18.34	3	Blood	Australia	Susceptible	[46]

ST – Sequence Type.

Since *C. glabrata* is known for the acquisition of point mutations leading to drug resistance phenotypes, we aimed to characterize the genomic variability of the isolates belonging to each resistance group and compared it to the variability observed in susceptible isolates. First, we determined the occurrence of SNPs and INDELs (Insertions and Deletions) by mapping the reads from each isolate to the *C. glabrata* reference genome. For subsequent analysis, only mutations occurring inside Open Reading Frames (ORFs) were considered in order to investigate a correlation between the acquisition of drug resistance and genomic variants in specific genes.

**Table 2.** Categorization of the isolates analyzed in this study according to their susceptibility profiles.

**Phenotype/Group**	**No of isolates**
Susceptible	22
Azole resistant	17
Echinocandin resistant	23
Echinocandin intermediate	18
Multi resistant	17

To determine which variants may be key to attain a resistance phenotype, the mutations commonly occurring in all isolates within each resistance group were compared to the mutations also taking place in susceptible isolates. No single mutation could be identified as present in all the resistant isolates in each group and in none of the susceptible isolates, indicating there is no individual mutation independently acquired by every resistant strain that solely justifies the resistance phenotype. We then set out to investigate mutations present in at least one isolate from each resistance group that are not present in any of the susceptible isolates, focusing on nonsynonymous mutations (**Table 3**).

**Table 3.** Nonsynonymous mutations occurring exclusively in at least one resistant isolate when compared to susceptible isolates.

**Phenotype/Group**	**No of mutations (absent in susceptible isolates)**
Azole resistant	6782
Echinocandin intermediate	5101
Echinocandin resistant	9070
Multi resistant	9201
Azole resistant	6782

**Figure 2:**
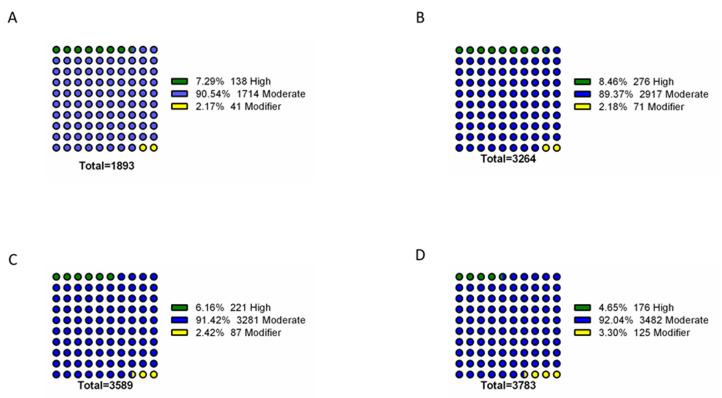
FIGURE 2: Impact analysis of genomic variants found exclusively in the genomes of resistant isolates. The assessed mutations are present in the genomes of azole resistant **(A)**, echinocandin intermediate **(B)**, echinocandin resistant **(C)** and multi resistant **(D)** isolates, being absent from the genomes of susceptible isolates. For each resistance group, the catalogued mutations are specific to that group. Variant impact was predicted with SnpEff [10]: High – the variant is assumed to have high (disruptive) impact in the protein, probably causing protein truncation, loss of function or triggering nonsense mediated decay; Moderate - A non-disruptive variant that might change protein effectiveness; Modifier – usually non-coding variants or variants affecting non-coding genes, where predictions are difficult or there is no evidence of impact.

**Figure 3:**
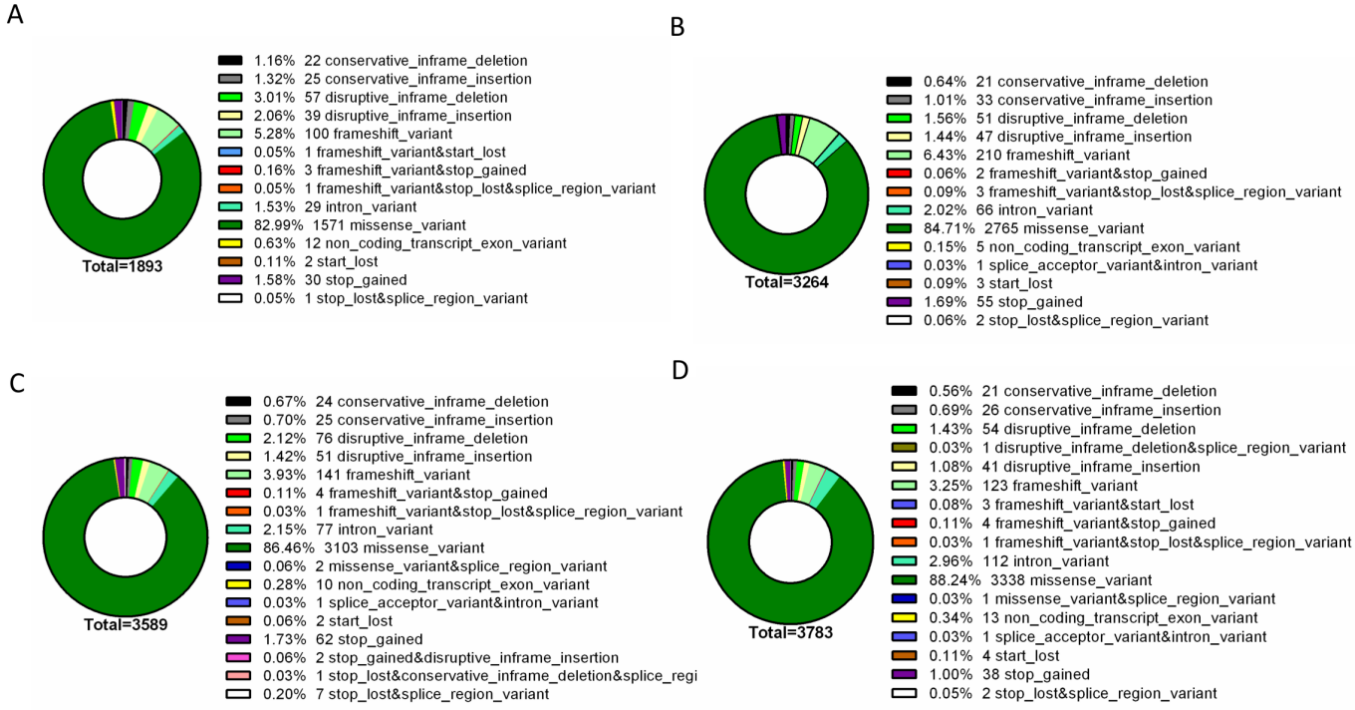
FIGURE 3: Type distribution of genomic variants found exclusively in the genomes of resistant isolates. The assessed mutations are present in the genomes of azole resistant **(A)**, echinocandin intermediate **(B)**, echinocandin resistant **(C)** and multi resistant **(D)** isolates, being absent from the genomes of susceptible isolates. For each resistance group, the catalogued mutations are specific to that group. Variant type was predicted with SnpEff [10].

To further identify phenotype-specific mutations, the dataset was subsequently filtered to only consider mutations specific to each resistance group (i.e., mutations absent from susceptible isolates and absent from other resistance groups). Overall, the final dataset comprised a range of 1893 – 3783 phenotype-exclusive nonsynonymous mutations (Table S1). These variants were grouped according to their predicted impact on the underlying gene (**Figure 2**). Mutations are considered of high impact when the variant is assumed to have high (disruptive) impact in the protein, probably causing protein truncation, loss of function or triggering nonsense mediated decay; and of moderate impact when the mutation is a non-disruptive variant that might change protein activity. Modifier mutations usually comprise non-coding variants or variants affecting non-coding genes, where predictions are difficult or there is no evidence of impact [10]. This last type of mutation constitutes the smaller part of the total mutations. Across all resistance groups, approximately 90% of the mutations were predicted to result in a moderate impact in the derived amino acid sequence, which includes conservative in frame deletions/insertions, disruptive in frame deletions/insertions or missense mutations. This distribution is consistent with the known prevalence of point mutations in *C. glabrata*, which is further reinforced by the predominance of missense mutations in the total variants pool (∼83-89%), followed by high impact frameshift variants (**Figure 3**). Despite this, only experimentally one could in-vestigate whether these mutations are GOF mutations or mutations that lead to the impairment of protein function.

### Mutations in genes commonly associated with resistance do not explain all the resistance phenotypes

A common approach in epidemiological and surveillance studies highlighting the emergence of azole resistance in *C. glabrata* isolates is to probe the *PDR1* gene sequence to correlate gene variants with azole resistance phenotypes. Similarly, echinocandin resistance is often correlated to *FKS1/FKS2* gene sequence polymorphisms. However, the variability in *PDR1* or *FKS* gene sequences in susceptible isolates is largely unknown, making it difficult to ascertain the likelihood of the observed mutations to have a causal impact in the phenotypic change.

**Figure 4:**
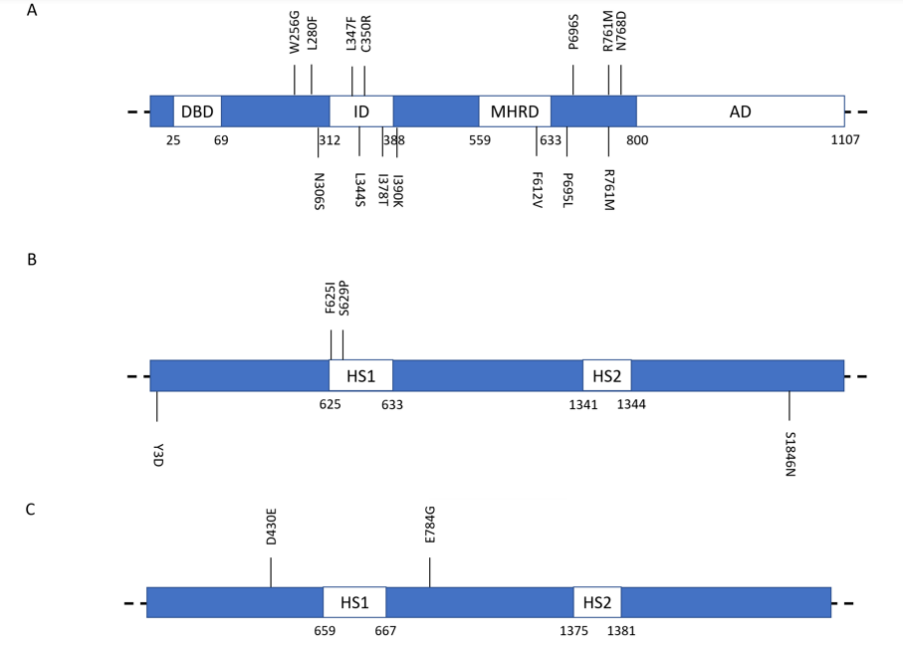
FIGURE 4: Representation of the variants found in resistance genes typically subjected to mutations conferring resistance to azoles or echinocandins. Protein domains are represented to provide visual illustration of the variants position in the protein context. **(A)** Upper mutations – *PDR1* variants identified in the genomes of azole resistant isolates but not in the genomes of susceptible isolates; Bottom mutations – *PDR1* variants identified in the genomes of multi resistant isolates but not in the genomes of susceptible isolates. **(B)** Upper mutations – *FKS1* variants identified in the genomes of echinocandin resistant isolates but not in the genomes of susceptible isolates; Bottom mutations – *FKS1* variants identified in the genomes of multi resistant isolates but not in the genomes of susceptible isolates. **(C)** *FKS2* variants identified in the genomes of echinocandin resistant isolates but not in the genomes of susceptible isolates. DBD – DNA Binding Domain, ID – Inhibitory Domain, MHRD – Middle Homology Regulatory Domain, AD – Activation Domain, HS1 – Hotspot 1, HS2 – Hotspot 2.

The genome sequencing data of multiple isolates was used to analyze the *PDR1* gene variants in azole resistant isolates and compare them with existing variants in susceptible strains. This approach enables the identification of the mutations that most likely underscore azole resistance, while simultaneously providing an estimate on the prevalence of mutations occurring in Pdr1 specifically in resistant isolates. Altogether, our variant analysis detected seven *PDR1* mutations exclusive of azole resistant strains, and each mutation was traced back to one single isolate (**Figure 4A**, Table S2). Two of these mutations are located within the inhibitory domain of the transcription factor (L347F, C350R). These results enabled the identification of a core set of Pdr1 mutations potentially driving azole resistance, although none could be detected to occur with higher frequency than the rest. Moreover, the data indicates that ∼41% of the analyzed azole resistant isolates contain Pdr1 mutations not present in susceptible strains, hinting that additional pathways or genomic variation may play a role in the acquisition of azole resistance in the clinical setting. Additionally, six out of the seven Pdr1 mutations are also absent in the isolates belonging to other resistance groups, reinforcing the notion that these variants are specific to the acquisition of azole resistance. From the indicated *PDR1* mutations, only L344S, found in a multi resistant isolate, has been experimentally demonstrated to result in a hyperactive form of Pdr1. Complementation of a Δ*pdr1* mutant strain with *PDR1*^*L344S*^ allele rendered *C. glabrata* cells more resistant to azoles when compared with complementation of the same mutant strain with the wild-type *PDR1* allele [11, 12]. Also, the transcript levels of *PDR1* and *CDR1* in the strain harboring the L344S mutation were higher compared with other azole resistant strains carrying *PDR1* hyperactive forms [12]. L344S mutation was found in multi resistant isolates. Remarkably, *PDR1*^*L344*^ allele was also found to promote ∼1.7 times the adherence of the wild-type strain, associated with increased expression of *EPA1* [12].

A similar analysis was carried out to probe the occurrence of mutations in the echinocandin targets *FKS1/FKS2*. Two *FKS1* mutations and two *FKS2* mutations were identified exclusively in echinocandin resistant isolates when compared to susceptible ones (**Figure 4B-C**, Table S2). The two Fks1 mutations are located within the hotspot region 1 (F625I, S629P). Additionally, all four variants were also absent from the isolates belonging to the other resistance groups and were traced back to four isolates in an asymmetrical manner: one of the *FKS1* mutations and one of the *FKS2* mutations were present in the same two closely related strains (WER17 and WER18; **Figure 1**); while the remaining two variants were traced back to two distinct strains (OER1 and WER14). As such, within the group of echinocandin resistant isolates, only ∼17% of the analyzed strains contain exclusive *FKS1/FKS2* mutations, indicating that echinocandin resistance can arise via other mechanisms and/or genomic variations. This is in strong contrast with the group of echinocandin intermediate isolates, where only one exclusive mutation from one single isolate was found in *FKS2*.

The emergence of multidrug resistance has been most frequently reported among *C. glabrata* isolates [13–16]. In this analysis, we also investigated *PDR1* and *FKS1/FKS2* variations in strains resistant to both azoles and echinocandins (not occurring in susceptible strains) and compared them with those from strains resistant to either azoles or echinocandins only. This analysis attained seven *PDR1* mutations and two *FKS1* mutations occurring in multi resistant strains (**Figure 4A-C**, Table S2), being all exclusive to this resistance group except for one of the Pdr1 mutations, which was commonly found in the isolate JMR1 and its paired isolate JAR1 from the azole resistance group (**Figure 1**). In fact, specific *PDR1* mutations in *C. glabrata* have been associated with adaptation to echinocandin exposure by enhancing adhesion to epithelial cells through increased expression of the epithelial adhesin gene *EPA1* [17]. The fact that *PDR1* polymorphisms found in multi resistant strains are distinct from those found in azole resistant strains strengthens the lack of targeted variants to a specific residue or protein domain. Two of the Pdr1 mutations occur within the inhibitory domain (L344S, I378T), while one is located within the middle homology domain (F612V; **Figure 4A**). As for the two Fks1 mutations taking place in this group, both traced back to a single isolate and are located outside of either hotspot region (**Figure 4B**).

### Genome wide variants to predict additional drug resistance determinants

The SNPs and INDELs occurring throughout the genomes of the analyzed isolates were further explored to investigate variants in additional genes that could contribute to each resistance phenotype. As stated before, we identified between 1893 and 3783 nonsynonymous variants exclusive to the genomes of resistant isolates (Table S1). As expected, the genes with higher number of polymorphisms correspond to adhesin encoding genes. This represents a significant constrain to achieve unbiased SNP/INDEL analysis in genome-wide studies, as adhesins are located in sub telomeric regions characterized by high plasticity [18, 19]. Most adhesin genes also have high content of repetitive regions [18, 19], which represents an adversity to read mapping algorithms and hinders reliable variant calling. Even so, adhesins have already been related with drug resistance in *C. glabrata* [20]. Therefore, the establishment of correlations between adhesin gene variants and possible biological outcomes needs to be taken cautiously.

Gene ontology (GO) term based functional characterization of the mutated genes in each resistance group is present in Table S3. The distribution appears to be focused on categories associated with ATP binding, transcriptional regulation, and phosphorylation. This enrichment possibly illustrates the acquisition of polymorphisms in genes from signal transduction pathways and/or regulatory networks. The role of GOF mutations in transcriptional regulators is well established in *C. glabrata* and other *Candida* species [21–23]. It seems reasonable to hypothesize that similar activity changing mutations in post-translation modification-related enzymes, such as kinases, can have a relevant impact as well.

Due to the emergence of azole resistance in *C. glabrata*, we explored the genome-wide variants data on this resistance group to highlight polymorphisms that can impact azole resistance and guide further experimental work to assess their biological impact. A number of genes involved in the ergosterol biosynthesis pathway are present in this group, including *ERG2, ERG3, ERG9, ERG20*, and *ERG28*. Particularly compelling is the presence of a mutation in *ERG3* resulting in the insertion of a stop codon (Y170*). Erg3 is responsible for the production of toxic sterols that incorporate and permeabilize the membrane during azole exposure [24]. Loss of function (LOF) mutations in this gene could represent an alternative pathway to mediate azole resistance, as described in other *Candida* species [25, 26]. Additionally, a missense mutation (E619Q) was found in the ergosterol biosynthesis and sterol uptake regulator Upc2A. This TF is a determinant of azole resistance by upregulating the azole target *ERG11* [27] and an engineered Upc2A GOF resulted in increased azole resistance [28]. Acquisition of Upc2 mutations was reported in azole resistant clinical isolates of *C. albicans* [29]; here we show that Upc2A variants can also be acquired *in vivo* by azole resistant *C. glabrata* isolates as a mechanism to achieve the resistant phenotype.

SNPs have been found in other regulators of azole resistance, including a missense mutation in Stb5 (N60Y). *STB5* is a negative regulator of azole resistance [30] and the identified mutation spans its DNA binding domain, which could result in impaired target recognition. Another potentially impacting variant is an amino acid duplication event in Gal11A (N111dup). Gal11A is a component of the mediator complex required for the correct regulatory activity of Pdr1 and demonstrated to be required for azole resistance [31].

Lastly, we searched for genes specifically mutated in resistant isolates and not in susceptible ones. Our analysis identified two genes mutated in azole resistant strains, one gene mutated in echinocandin resistant strains, three genes mutated in echinocandin intermediate strains, and two genes mutated in multi resistant strains (**Table 4**). From those, we analyzed six genes for a potential role in *C. glabrata* drug resistance. As they lack functional characterization in *C. glabrata*, their closest *S. cerevisiae* orthologs were investigated to make a preliminary prediction on the relevance of these genes. Indeed, most of them have been described to influence susceptibility to a number of xenobiotics in *S. cerevisiae*. From the dataset, *CAGL0I00352g* (Sc*RAD6*) is the only gene mutated in more than one resistance group, being common to azole resistant and multi resistant isolates. However, the identified mutation was found in both azole resistant (WAR9) and multi resistant (WMR3) strains, which are closely related and may represent paired isolates (**Figure 1**). In the yeast model, *RAD6* confers resistance to a wide variety of compounds, is involved in ubiquitination, chromosome stability and genome rearrangements [32]. Given that *C. glabrata* is known for rapidly acquiring resistance to drugs and genome rearrangements being common among clinical isolates, this gene could represent a promising target for further studies. Using the PathoYeastract database [33], *RAD6* was found to be positively regulated by Rpn4, a regulator recently found to mediate azole resistance [34], during fluconazole stress.

**Table 4.** Genes affected by nonsynonymous mutations occurring exclusively in resistant isolates when compared to susceptible isolates.

**Group**	***C. glabrata* gene**	***S. cerevisiae* homolog**
Azole resistant	*CAGL0I00352g*	*RAD6*
	*CAGL0I01430g*	*ANB1*
Echinocandin resistant	*CAGL0C04411g*	*HTA2*
Echinocandin intermediate	*CAGL0I06270g*	*QCR8*
	*CAGL0L06930g*	*YDL085C-A*
	*CAGL0M10241g*	*RPL14A*
Multi resistant	*CAGL0I00352g*	*RAD6*
	*CAGL0L12540g*	*EGD1*

**Figure 5:**
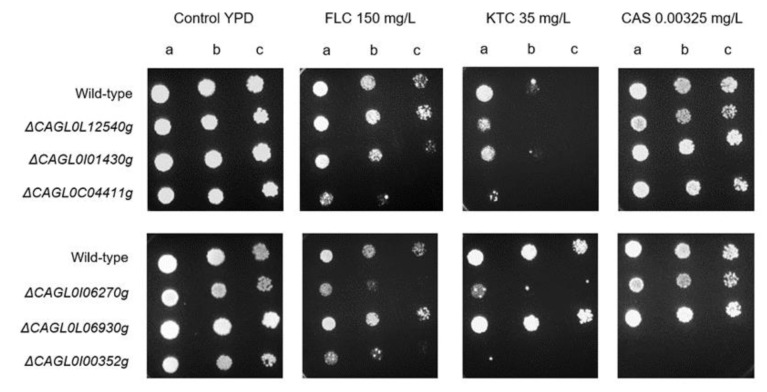
FIGURE 5: Comparison of the susceptibility to antifungal azole drugs and caspofungin, at the indicated concentrations, of the *C. glabrata* KEU100 wild-type strain or derived single mutant strains on YPD agar plates by spot assays. The inoculum was prepared as described in the Materials and methods section. Cell suspensions used to prepare the spots correspond to 1:5 (b) and 1:25 (c) dilutions of the cell suspensions used in (a). The displayed images are representative of at least three independent experiments.

In an attempt to evaluate whether the used large-scale genome sequencing comparison can indeed enable the identification of new players in drug resistance in *C. glabrata*, six out of the seven genes identified in **Table 4** were deleted in a wild-type parental strain and their ability to tolerate inhibitory concentrations of antifungal drugs assessed. Remarkably, consistent with the genome sequencing indications, susceptibility assays demonstrated that the *C. glabrata* deletion strain Δ*CAGL0I00352g* is indeed highly susceptible to the triazole fluconazole and to the imidazole ketoconazole, and fully susceptible to the echinocandin caspofungin (**Figure 5**). Interestingly, the lack of either *CAGL0I06270g* (*ScQCR8*) or *CAGL0C04411g* (*ScHTA2*) also rendered *C. glabrata* cells susceptible to azoles, but not to caspofungin (**Figure 5**). In *S. cerevisiae, ScHTA2* is a core histone protein required for chromatin assembly and chromosome function and a deletion mutant strain was indeed found to be susceptible the agricultural azole penconazole, when compared to the wild type [35]. On the other hand, *ScQCR8* encodes the subunit 8 of ubiquinol cytochrome-C reductase (Complex III), which is a component of the mitochondrial inner membrane electron transport chain and had not yet been reported to be involved in drug resistance.

### Genome plasticity across *C. glabrata* isolates - copy number variations and aneuploidies

The haploid nature of *C. glabrata* makes it prone to acquire polymorphisms, but this pathogen can also tolerate high genome plasticity such as structural rearrangements. Here we used genome sequencing data to evaluate genome plasticity and analyze copy number variants (CNV) across multiple *C. glabrata* clinical isolates.

CNVs were assessed based on a depth of coverage approach to detect genomic segments with higher or lower copy numbers. Overall, we detected events of copy number variation in 24 (24.7%) isolates, regardless of resistance group (Figure S1). Of those, the most prominent detected changes were aneuploidies involving whole duplications of chromosome E in 15 (62.5%) strains, and partial deletions or duplications in chromosome M in six (25%) strains. Other structural changes comprised aneuploidies involving whole duplications of chromosomes A or C, and partial duplications in chromosomes B or G (Figure S1).

No discernible pattern could be identified between CNVs and phenotypic outcome. The most prevalent CNVs detected (ie. chromosome E duplications) affect the copy number of the azole target encoding gene *ERG11*, but this aneuploidy is present in isolates from both susceptible and resistant strains. Despite this, experimental analysis on chromosome E alterations would be interesting since it affects *ERG11* copy number. Likewise, other structural variants affecting resistance genes, such as increased copy number in the first half of chromosome A (*PDR1*) or the first half of chromosome M (*CDR1*), could not be associat-ed exclusively with azole resistant strains. No relevant CNVs were found in regions spanning the echinocandin resistance genes *FKS1/2*. The data appears to highlight that some chromosomal segments are more prone to CNVs in the *C. glabrata* genome, but a correlation with a particular phenotype is unclear.

### Isolates 50570 and 73281 display unconventional genetic basis for acquired azole resistance

In this study, the genomes of *C. glabrata* isolates 50570, 67367 and 73281, collected from patients admitted to a tertiary care center in Portugal, were sequenced. The multi strain analysis performed in this work allowed to characterize these isolates in a broader context and compare them with other *C. glabrata* isolates collected worldwide.

The three isolates are categorized as azole resistant, and their genomes were investigated to provide clues on the genetic basis behind their phenotype. None of the isolates showed aneuploidies or CNVs across their genomes. Only isolate 67367 acquired a nonsynonymous SNP in Pdr1 (C350R) that was not present in susceptible isolates. Isolate 50570 harbors Pdr1 SNPs that are also present in susceptible isolates, while isolate 73281 has no Pdr1 SNPs, when compared to the reference strain.

The absence of specific Pdr1 mutations in isolates 50570 and 73281 led us to screen the genome variants acquired by these strains to identify the potential genetic basis for their azole resistance phenotype. Only variants absent from the other resistance groups were considered. Isolate 50570 possesses 149 exclusive mutations distributed across 109 genes, while isolate 73281 possesses 172 exclusive mutations across 143 genes (Table S4).

**Table 5.** Genes investigated for a potential role in *C. glabrata* drug resistance, based on the genome-wide analysis of the azole resistant isolates 50570 and 73281.

***C. glabrata* gene**	***S. cerevisiae* homolog**	**Selection criteria**
*PEX11*	-	Peroxisomal activity
*CAGL0A04543g*	*AAR2*	Altered azole susceptibility/differential expression
*CAGL0G04169g*	*YDR306C*	Frameshift mutation
*CAGL0G04455g*	*IME2*	AmB resistance
*CAGL0H09130g*	*MNN4*	Frameshift mutation
*KES1*	-	Ergosterol biosynthesis
*CAGL0F03003g*	*HKR1*	Frameshift->stop
*CAGL0H03465g*	-	Frameshift mutation
*CAGL0I09878g*	*FAA1*	Lipid metabolism
*CAGL0J00891g*	*LTV1*	Altered azole susceptibility/differential expression
*CAGL0J08613g*	*YVC1*	Frameshift mutation

**Figure 6:**
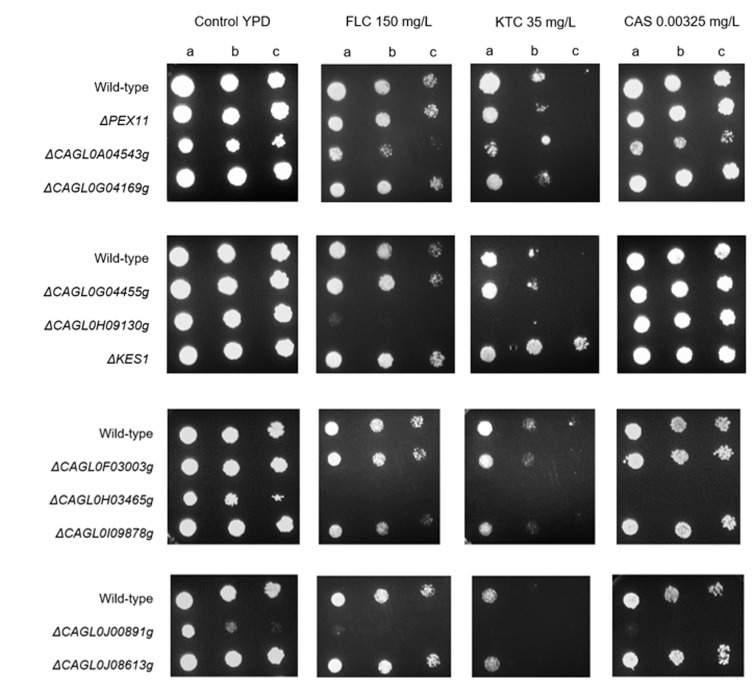
FIGURE 6. Comparison of the susceptibility to antifungal azole drugs and caspofungin, at the indicated concentrations, of the *C. glabrata* KEU100 wild-type strain or derived single mutant strains on YPD agar plates by spot assays. The inoculum was prepared as described in the Materials and methods section. Cell suspensions used to prepare the spots correspond to 1:5 (b) and 1:25 (c) dilutions of the cell suspensions used in (a). The displayed images are representative of at least three independent experiments.

Additionally, we decided to investigate the role of some other of those potentially interesting genes reported in Table S4. We have not performed an exhaustive analysis, but we rather chose eleven genes (excluding adhesin-like proteins and genes that had already been reported to be associated with *C. glabrata* azole/echinocandin resistance) to investigate a potential role in *C. glabrata* multi drug resistance (**Table 5**). We tested those eleven potentially interesting genes by comparing the susceptibility of each single deletion mutant strain with the wild-type strain towards fluconazole, ketoconazole and caspofungin resistance (**Figure 6**).

Searching isolate 50570 for mutations impacting genes previously reported to alter susceptibility to azole drugs, we found a non-synonymous SNP (nsSNP) in the TF *DAL81* (*CAGL0F06743g*) and a nsSNP in the cell surface integrity gene *KEX2* (*CAGL0J07546g*) [36, 37]. Interestingly, a nsSNP was found in *KES1* (*CAGL0M02431g*), encoding an ergosterol biosynthesis enzyme, which can account for a more direct role in mediating azole resistance. Nonetheless, the single deletion strain did not present azole susceptibility (**Figure 6**). On the other hand, isolate 73281 was found to harbor a nsSNP in the component of the mediator complex *NUT1*, which has been demonstrated to alter fluconazole resistance [38], probably by influencing interactions with other mediator components and transcriptional programs. In fact, mediator complex components interacting with Pdr1 can be required for full azole resistance, as observed for Gal11 [31]. Genes that have been associated with azole susceptibility phenotypes found to harbor point mutations also include the cell wall biosynthesis gene *KTR2* and the V-ATPase assembly gene *VPH2*, which has been demonstrated to be required for antifungal drug resistance in *C. glabrata* [39]. Moreover, a nsSNP was also found in the TF *CAGL0L04576*, encoding an ortholog of the *S. cerevisiae* multidrug resistance regulator *YRM1*. Additionally, this isolate also acquired a missense mutation in the non-cytoplasmic domain of the sterol importer Aus1, an ABC transporter that functions as sterol importer and contributes to azole resistance [40].

Interestingly, both *CAGL0H03465g* and *CAGL0J00891g* encoding genes appear to be essential for *C. glabrata* normal growth, since the single deletion strains present hampered growth under control conditions (**Figure 6**). Nonetheless, it is clear that the single deletion strains are much more susceptible to both azoles and echinocandins, when compared to the parental strain (**Figure 6**). On one hand, *CAGL0J00891g* encodes a putative protein required for growth at low temperature that curiously was found to be downregulated in an azole-resistant strain [23]. On the other hand, *CAGL0H03465g* encodes a protein of unknown function and has no homolog in the closely related *S. cerevisiae*. *C. glabrata* Δ*CAGL0A04543g* cells also present growth difficulties, but not as much as either *CAGL0H03465g* or *CAGL0J00891g*, and so it is not possible to distinguish the growth defect caused by azoles or echinocandins from that exhibited under control conditions. Interestingly, *CAGL0A04543g* encodes a putative component of the U5 snRNP and was also found to be downregulated in an azole-resistant strain [23].

On the other hand, presenting normal growth under control conditions, Δ*CAGL0H09130g* mutant cells were found to be fully susceptible to azoles (**Figure 6**). Based on the function of its *S. cerevisiae* ortholog *ScMNN4, CAGL0H09130g* encodes a putative positive regulator involved in mannosylphosphorylation of N-linked oligosaccharides that may also function as a mannosylphosphate transferase.

Although the remaining selected genes have not presented any significant drug resistance-associated phenotype, the results obtained reinforce the notion that genome-wide analysis is a strong and valuable approach to gain new knowledge on alternative mechanisms of acquired drug resistance in clinical isolates. Of course, additional potentially interesting players reported in Table S4, and not tested in this study, would also be worth evaluating in the future.

### Conclusions

Genome sequencing is a powerful tool that contributes to the advancement of drug resistance studies, population dynamics and evolution in fungal pathogens. Therapy regimens in the clinical setting can now be adjusted according to the genetic background in resistance genes, where isolates can be categorized as wild type of non-wild type in relevant *loci*.

*Candida* species show remarkable population diversity and genome plasticity, meaning it can be difficult to associate specific genomic variants to a particular phenotype. Here, we analyze the genomes of globally distributed *C. glabrata* isolates with various antifungal susceptibility profiles. The isolates are phylogenetically grouped according to their sequence types and independently of their resistance group of collection site. A comprehensive analysis of the genomic variations occurring in each resistance group of isolates led to the identification of genetic variants specific to each drug resistance profile. The data concludes that changes in the most studied drug resistance genes only occur in a fraction of the resistant isolates. The genomic landscape was further explored to identify additional genetic basis of resistance, both at the nucleotide or chromosome level. The strategy showcased in this study exemplifies how high throughput sequencing data, associated with drug susceptibility information, can be used to narrow down the possible genetic variants leading to a resistant phenotype. The preliminary phenotypic evaluation obtained for deletion mutants on some of the identified genes further shows how these results can be used to guide the identification of new players responsible for the clinical acquisition of drug resistance and the underlying genetic mechanisms.

## MATERIALS AND METHODS

### Sequencing Data and Genome Assembly

In this study, isolates 50570, 67367 and 73281 were sequenced as described previously [41] and their susceptibility profiles determined by Minimum Inhibitory Concentration (MIC) assays according to EUCAST guidelines [42, 43]. Raw sequencing data was deposited under BioProject PRJNA738673. Raw sequencing reads from additional *C. glabrata* clinical isolates were retrieved from publicly available BioProject submissions containing Illumina Whole Genome Sequencing (WGS) data generated from previous studies [41, 44–48]. Only isolates with known levels of susceptibility to antifungal drugs were considered. Categorization of each isolate was carried out using the established breakpoints by EUCAST (azoles, amphotericin B) or CLSI (echinocandins). In total, 97 *C. glabrata* clinical isolates were included in the analysis (**Table 1**). Reads for each isolate were trimmed with Trimmomatic v0.38 [49] to remove low-quality bases (PHRED score < 15) and pairs with reads shorter than 30 bases after trimming were excluded. SPAdes v3.12.0 [50] was used to assemble reads into contigs using the --careful option.

### Phylogenetic Analysis

The phylogenetic relationship among the 97 sequenced *C. glabrata* isolates was inferred with CSI Phylogeny 1.4 [51] based on the concatenated alignment of high-quality SNPs from genome assemblies for each isolate and the reference strain CBS138. Tree representation was performed with the Molecular Evolutionary Genetics Analysis (MEGA) v11 [52]. Multi Locus Sequence Typing (MLST) was performed for each isolate using six *loci* (*FKS, LEU2, NMT1, TRP1, UGP1, URA3*) and each sequence type (ST) identified according to the PubMLST database for *C. glabrata* [53].

### SNP Calling and Copy Number Variants

Reads were aligned against the reference assembly of the CBS138 strain [54] with the Burrows-Wheeler Aligner (BWA) v0.7.17 [55], using the BWA-MEM algorithm with default parameters. Single Nucleotide Polymorphisms (SNP) and Insertions and Deletions (INDEL) were identified using the Genome Analysis Toolkit (GATK) v4.0.8.1 [56] with an haploid model. Low-quality variants were filtered using BCFTools v1.9 [57] based on thresholds for mapping quality (> 30) and read depth (> 5). The attained variants were subsequently annotated using SnpEff v4.3 [10]. Copy number variations at genomic regions were determined from the expected depth of coverage. After mapping the reads of each isolate to the reference genome, a GATK pipeline to determine structural variants was used. Calling deletions or duplications was based on read counts at genomic regions and normalizing by the number of reads per region to attain denoised copy ratios. Allelic counts were then determined and used to call copy ratios across genomic segments.

### Strains and growth media

*C. glabrata* parental strain KUE100 [58] and all the derived deletion mutants were batch-cultured at 30°C, with orbital agitation (250 rpm) in Yeast Extract-Peptone-Dextrose (YPD) medium containing (per liter): 20 g glucose (Merck), 20 g Peptone (Merck) and 10 g Yeast extract (Merck).

### Disruption of *C. glabrata* ORFs

The deletion of the *C. glabrata* genes addressed in this study was carried out in the parental strain KUE100 using the method described by Ueno *et al.* [59]. Genes of interest were replaced by a DNA cassette including the *CgHIS3* gene, through homologous recombination. The PCR primers used to generate the replacement cassette for each gene and the primers used for PCR confirmation of gene deletion are present in Supplementary Table S5. The pHIS906 plasmid including *CgHIS3* was used as a template and transformation was performed as described previously [58].

### Antifungal susceptibility assays

*C. glabrata* cells susceptibility to toxic concentrations of the selected antifungal drugs was evaluated by spot assays. Cell suspensions used to inoculate agar plates were prepared with mid-exponential cells grown in YPD until an OD_600nm_=0.5±0.05, and then diluted in sterile water to obtain suspensions with an OD_600nm_=0.05±0.005. These cell suspensions and subsequent 1:5 and 1:25 dilutions were applied as 4 µL spots onto the surface of appropriate solid media supplemented with adequate concentrations of chemical stressors. The antifungal drugs tested included the following compounds, used in the specified concentrations: fluconazole 150 mg/L, ketoconazole 35mg/L and caspofungin 0.00325mg/L (all from Sigma).

## SUPPLEMENTAL MATERIAL

Click here for supplemental data file.

All supplemental data for this article are available online at https://www.microbialcell.com/researcharticles/2022a-pais-microbial-cell/.

## Abbreviations:

CNV: – copy number variant,
GOF: – Gain-Of-Function,
INDELs: – Insertions and Deletions,
nsSNP: – non-synonymous SNP,
SNP: – single nucleotide polymorphism,
TF: – transcription factor.
