# Supplementary material for: Multiple genome analysis of Candida glabrata clinical isolates renders new insights into genetic diversity and drug resistance determinants
Source: Microb Cell. 2022 Oct 13;9(11):174–89. doi: 10.15698/mic2022.11.786 (PMC9662024; doi:10.15698/mic2022.11.786)
Supplement: Supplementary file 1 [file mic-09-174-s01.zip › 2022A Pais Microbial Cell Supplementals/2022A Pais-supplementary-material.docx]

**Supplementary Material**

**
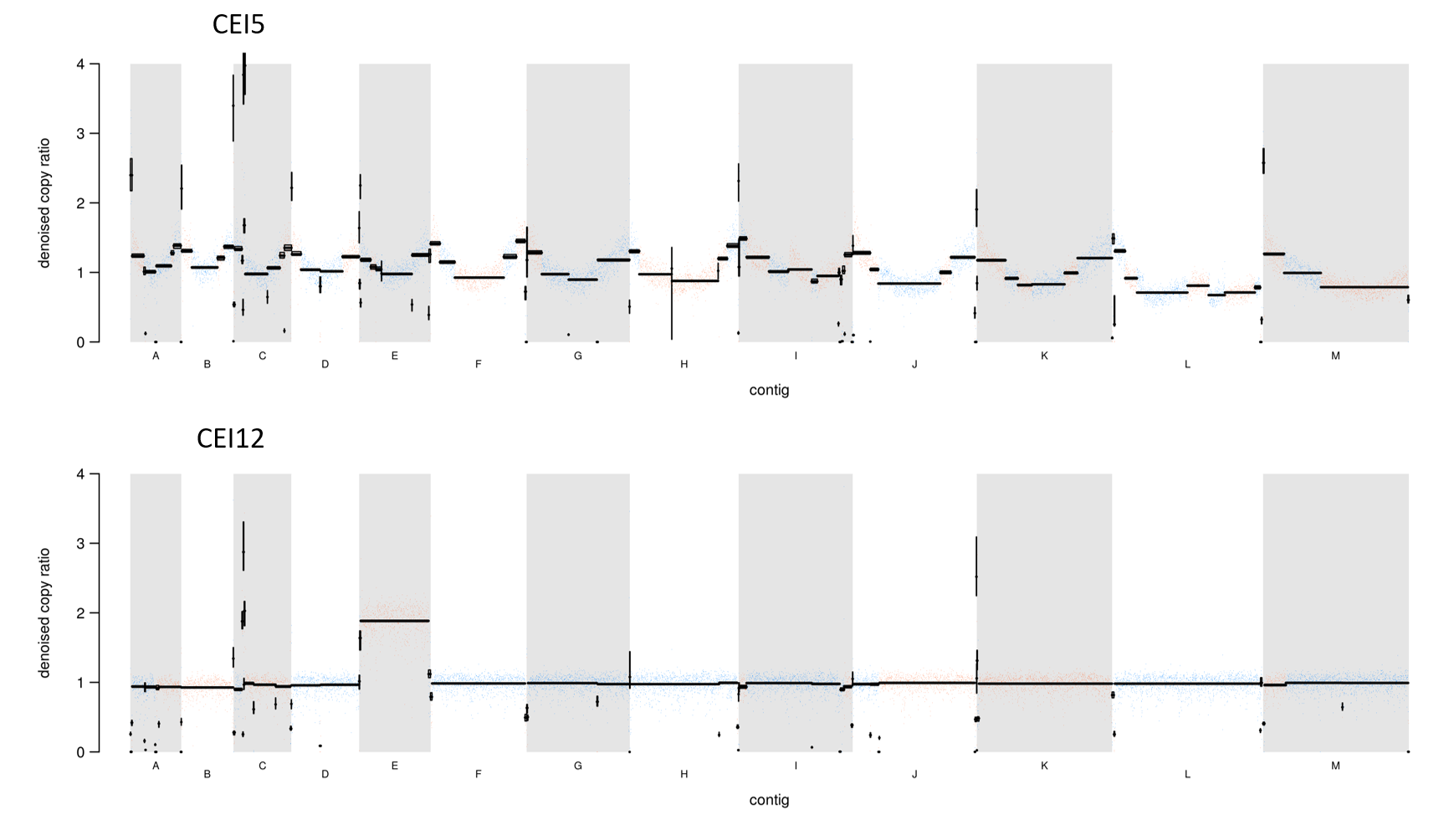
** **
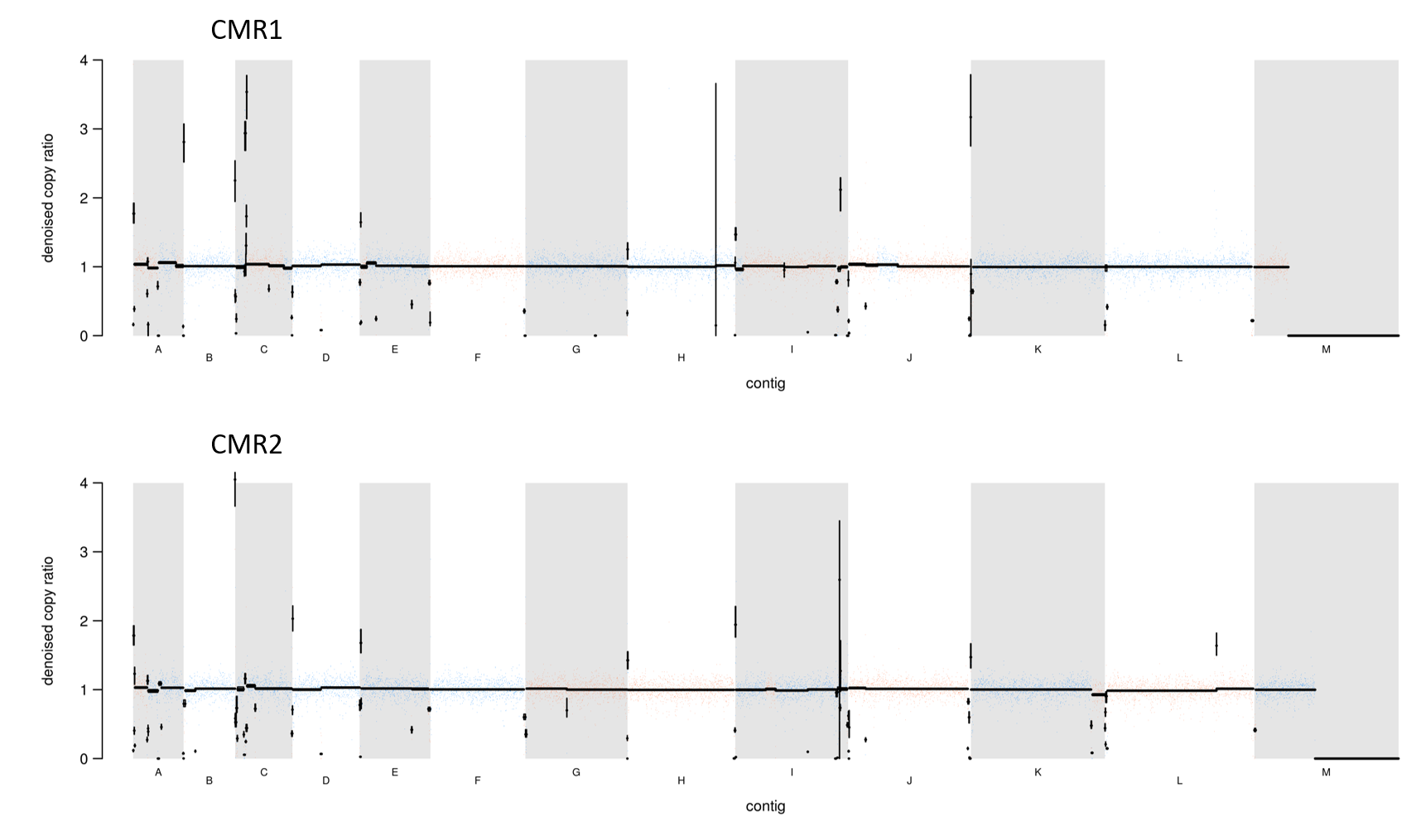
**
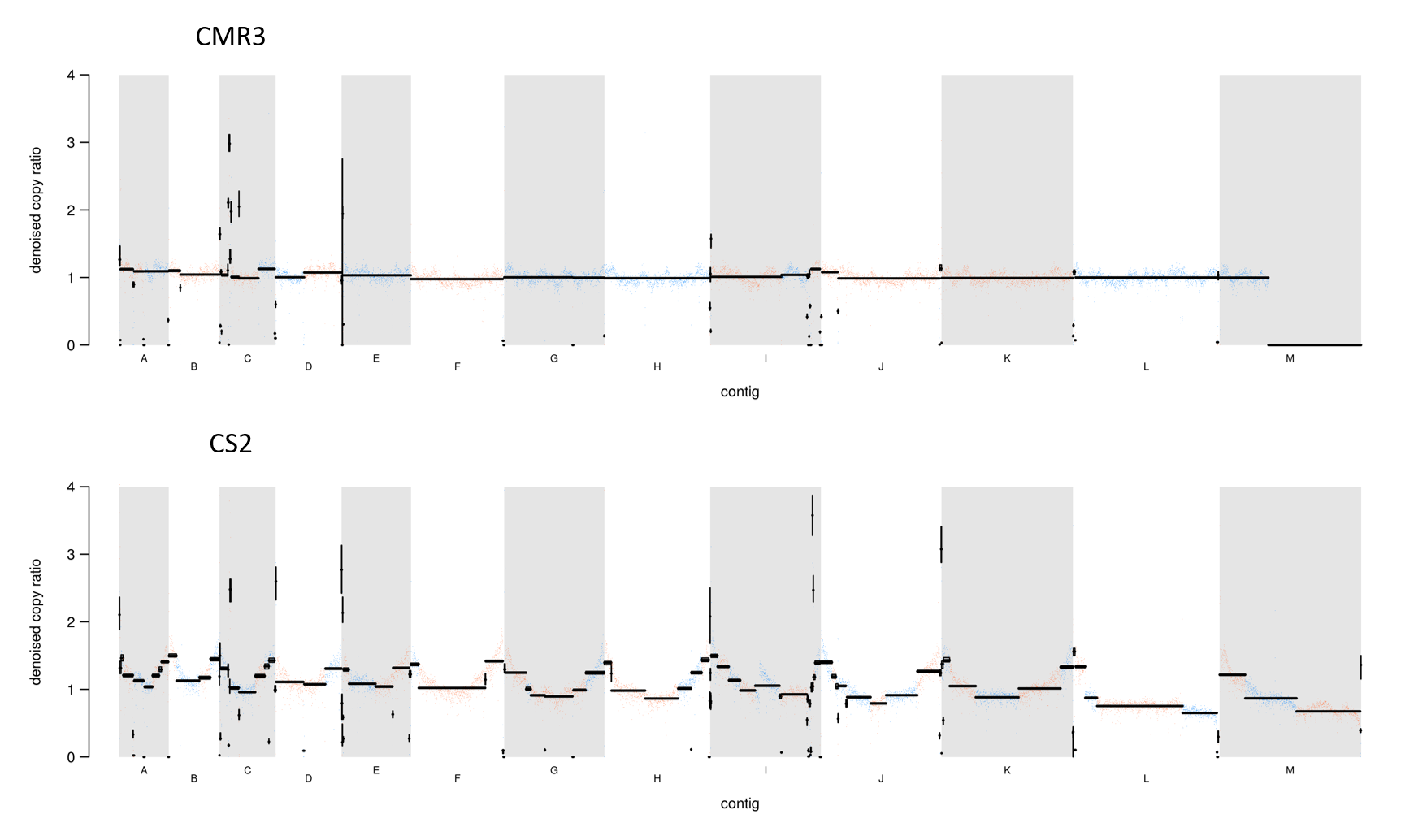

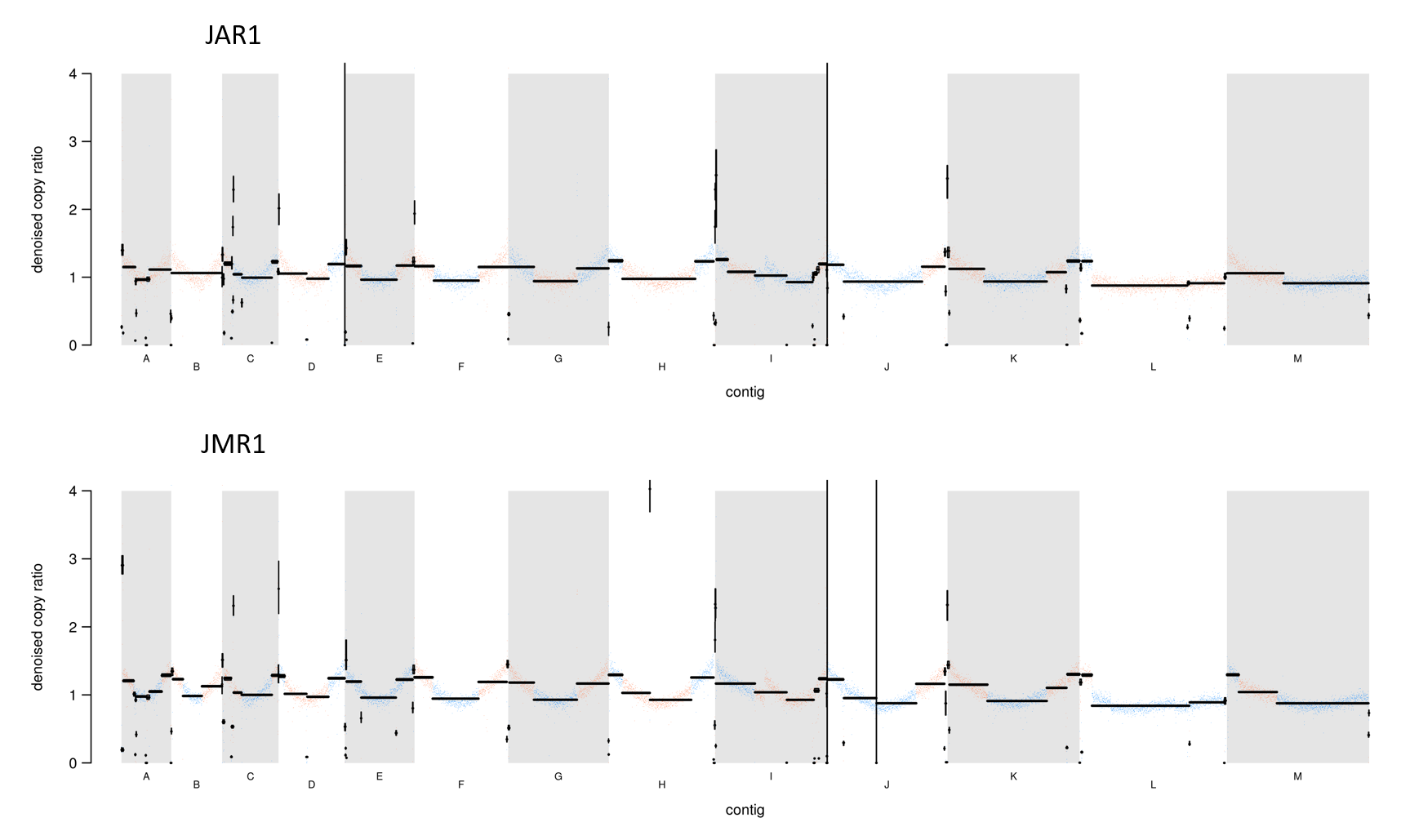

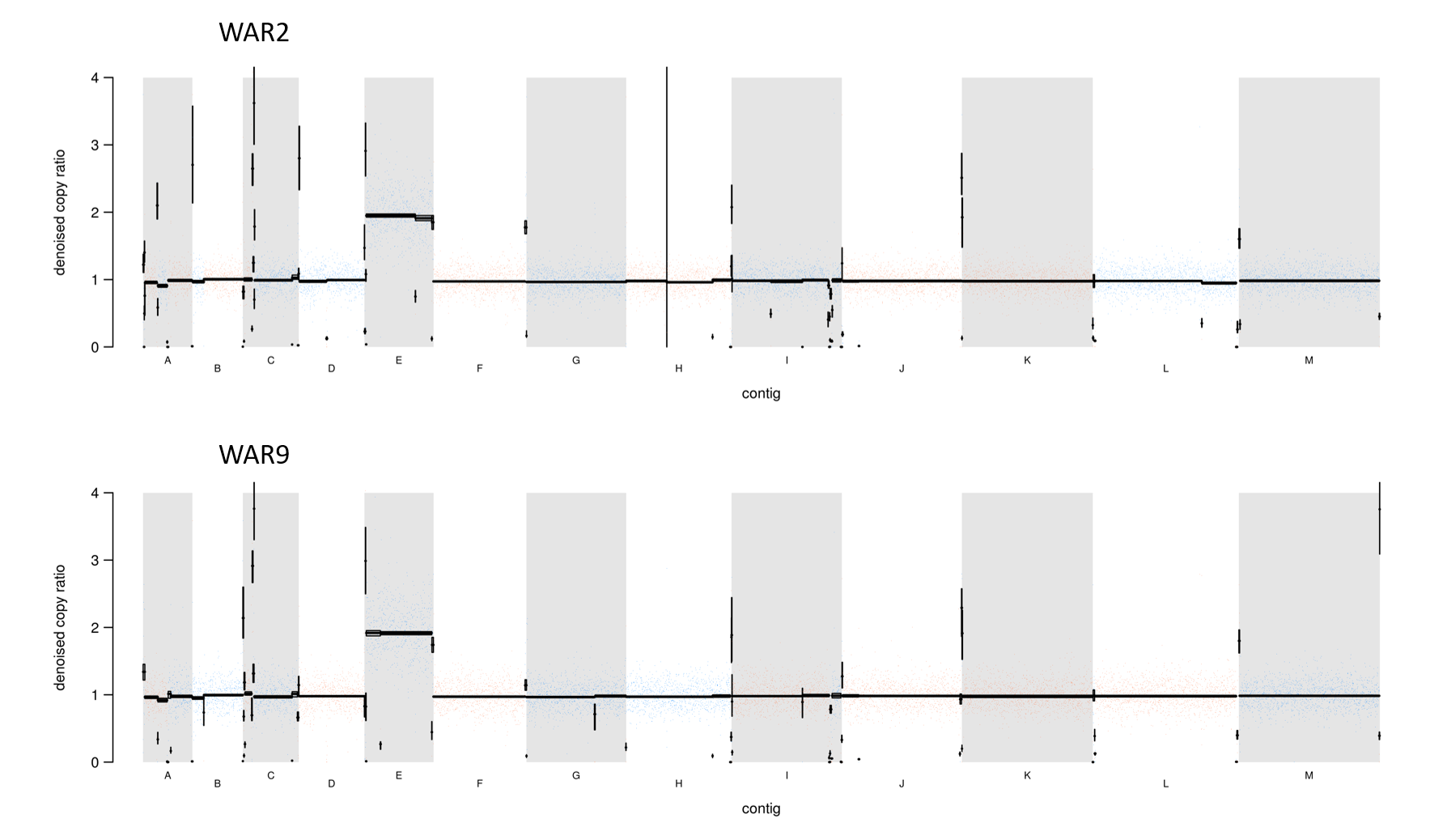

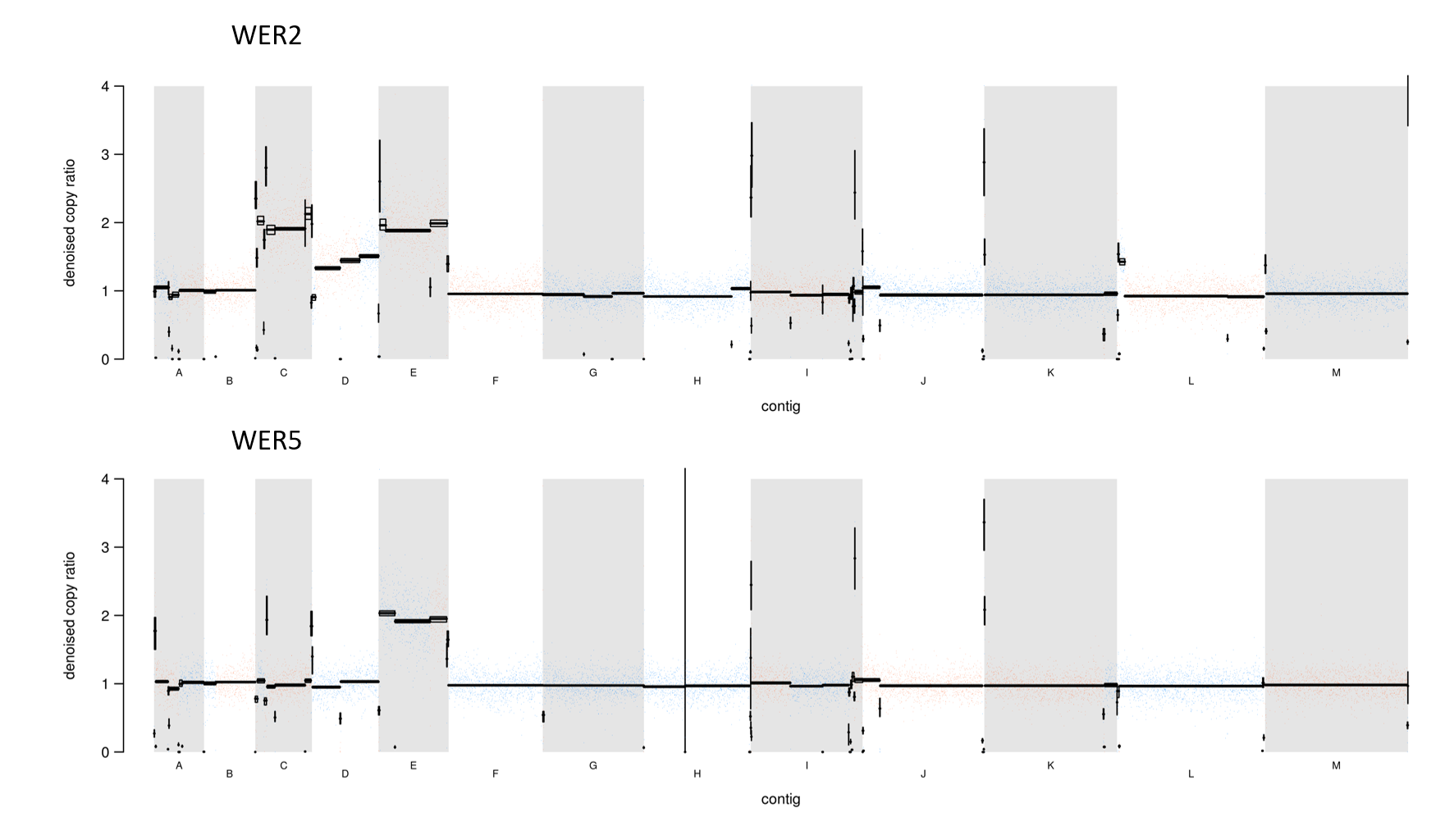

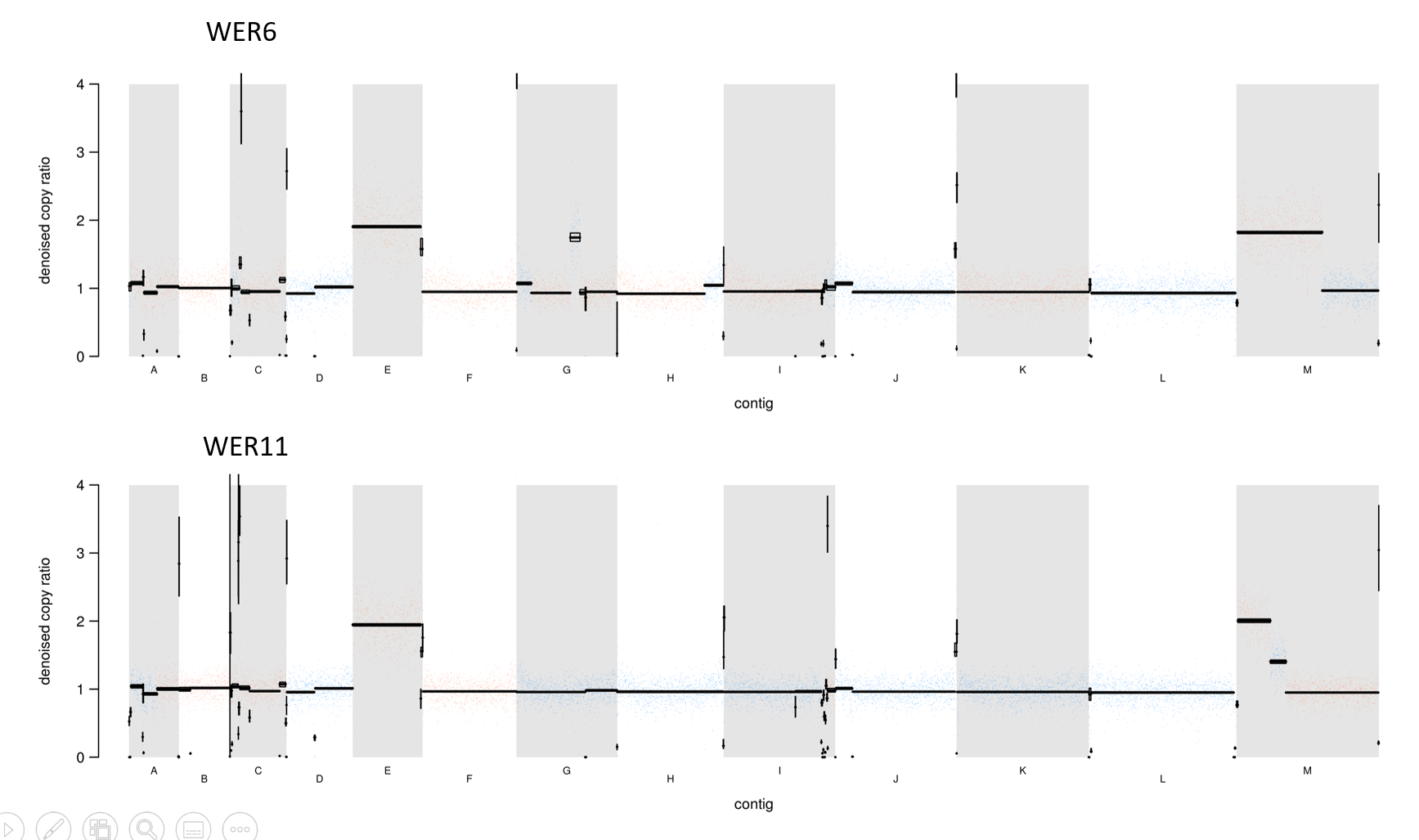

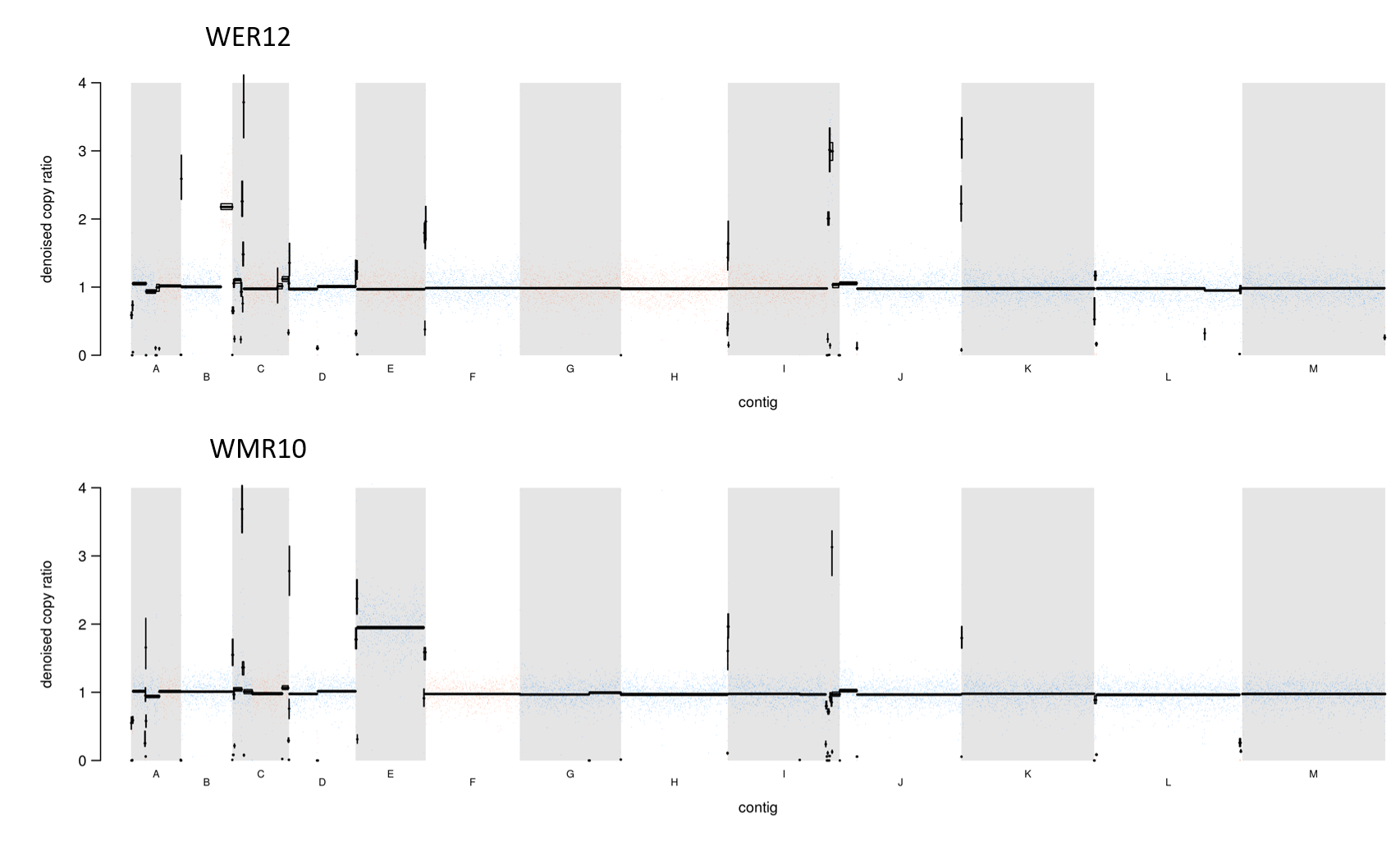

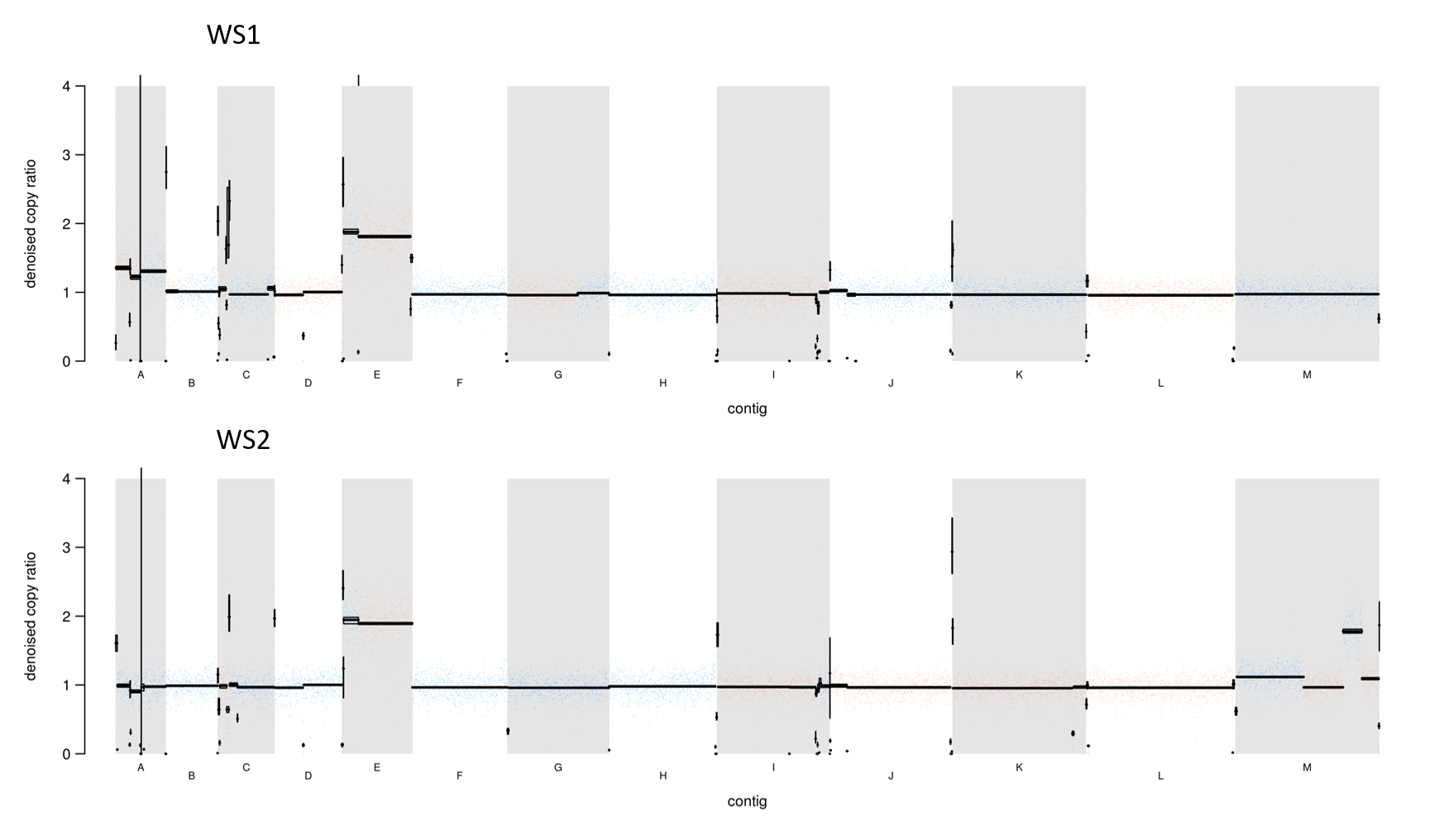

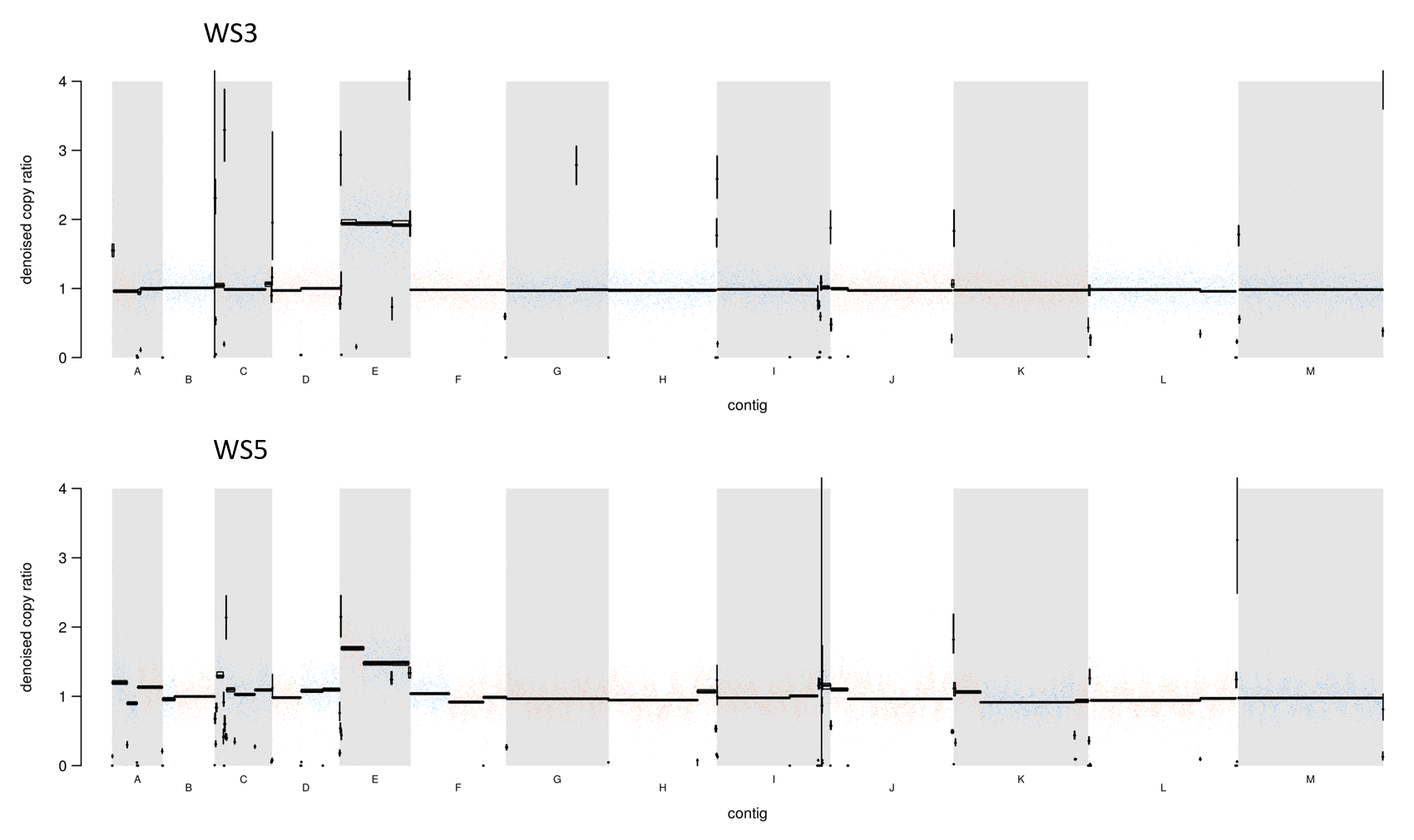

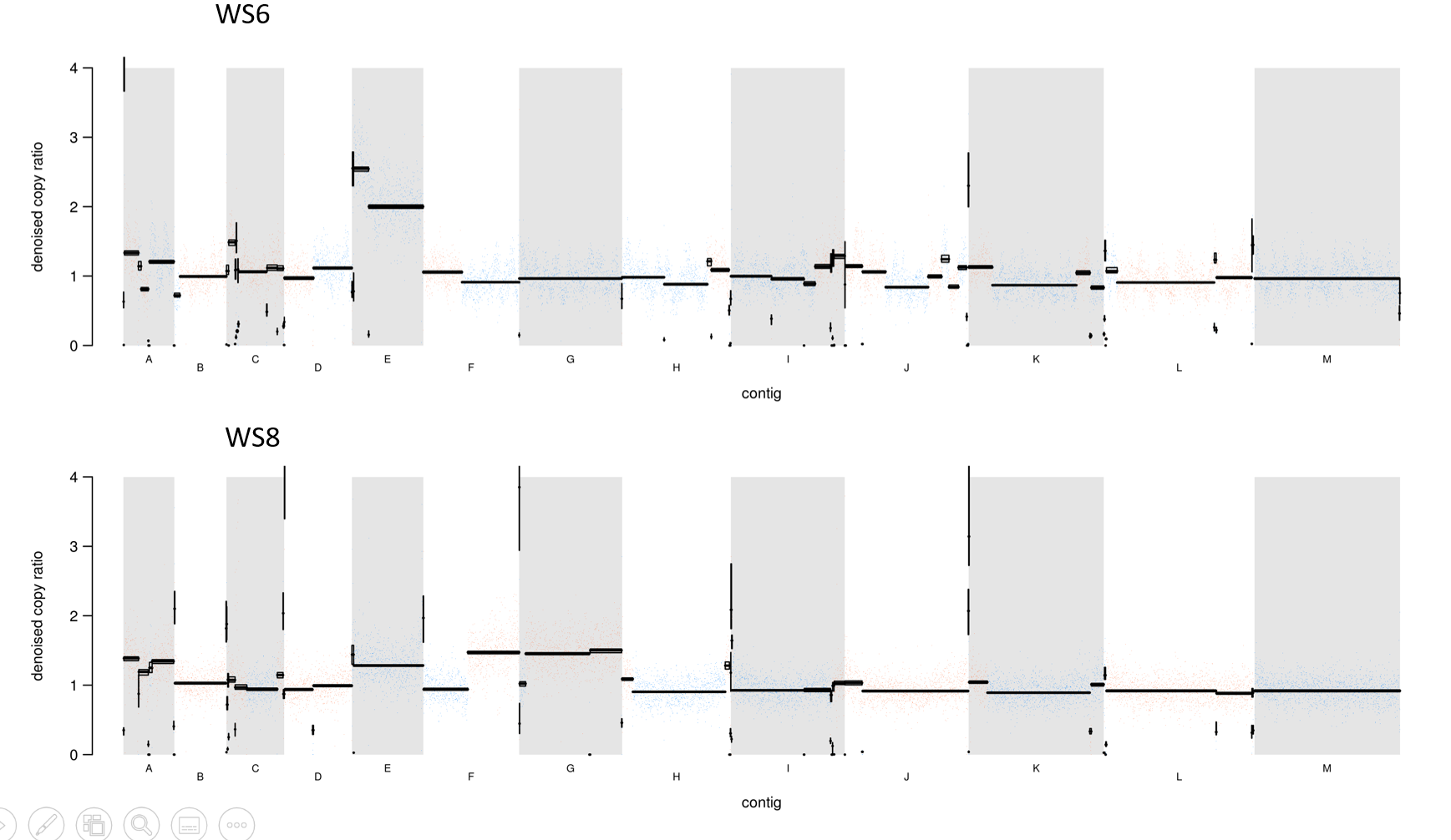

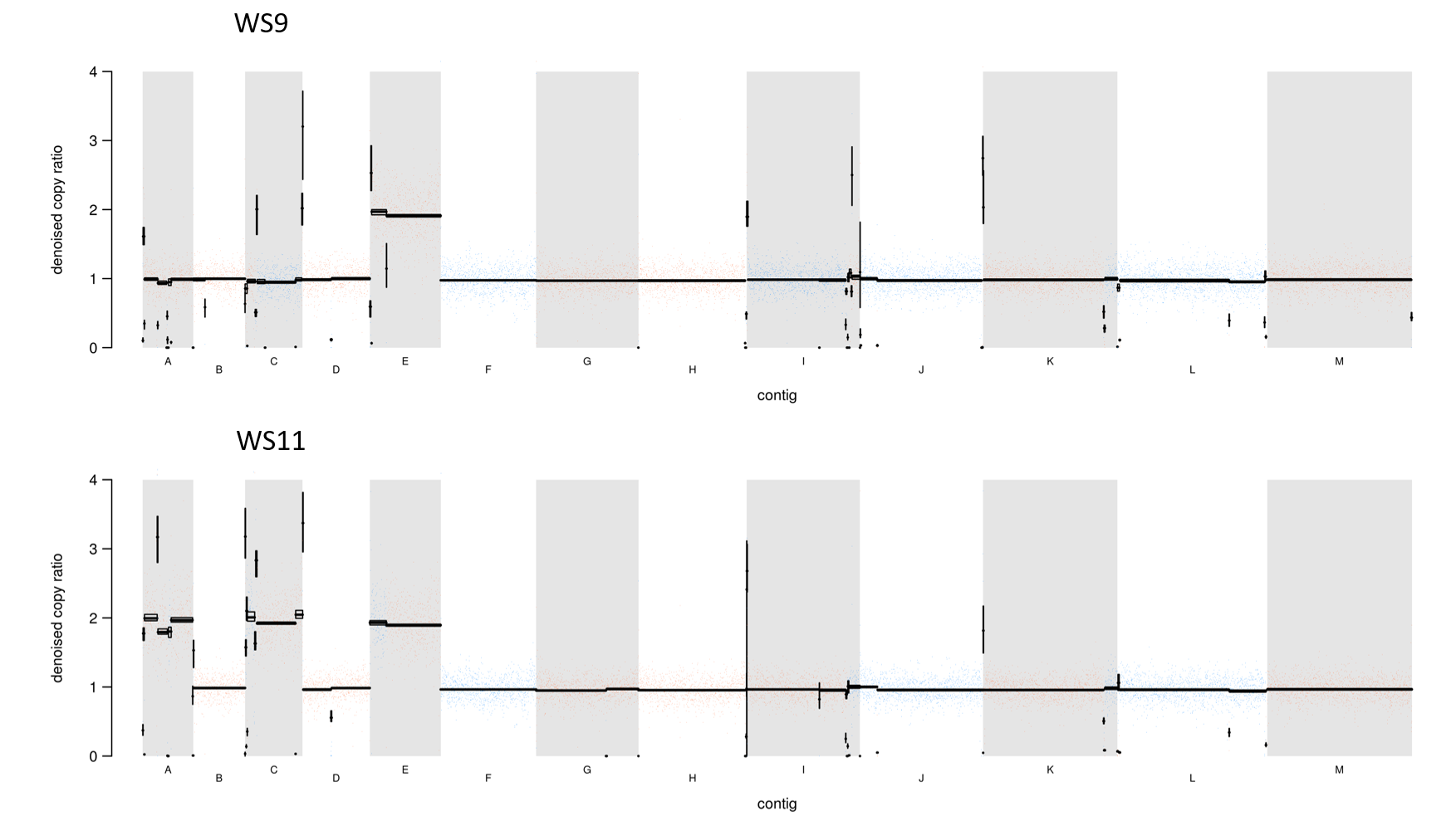


**Figure S1.**

**Supplementary Material Legends**

**Figure S1 –** Representation of copy number variants found in the genomes of *C. glabrata* isolates. Only isolates whose genome was found to harbor CNVs are shown. White and grey bars represent each chromosome, with the corresponding letter shown below. Denoised copy ratios are shown as points. The colors of the points alternate with the segmentation, different segments are indicated by alternating blue and orange color groups. The denoised median is drawn in thick black. Copy number variants were determined by a depth of coverage approach using GATK [25].

**Table S1 –** Exclusive nonsynonymous mutations in the genomes of each resistance group. The indicated variants are unique to each group, not being present in the genomes of susceptible isolates nor in the genomes of resistant isolates from other groups.

**Table S2 –** Exclusive nonsynonymous mutations found in *PDR1* or *FKS1/2* genes in the azole resistance, echinocandin resistance or multidrug resistance groups. The indicated variants are unique to each group, not being present in the genomes of susceptible isolates.

**Table S3 –** GO term categorization of the genes found to harbor exclusive nonsynonymous mutations in the genomes of each resistance group. Go analysis was performed at the Candida Genome Database.

**Table S4 –** Exclusive nonsynonymous mutations in the genomes of isolates 50570 and 73281. The indicated variants are unique to each genome, not being present in the genomes of susceptible isolates nor in the genomes of other resistant isolates.

**Table S5.** List of oligonucleotides designed and used in this study for gene disruption.
